# The Emerging Role of TR**α**1 in Cardiac Repair: Potential Therapeutic Implications

**DOI:** 10.1155/2014/481482

**Published:** 2014-02-09

**Authors:** Constantinos Pantos, Iordanis Mourouzis

**Affiliations:** Department of Pharmacology, University of Athens, 75 Mikras Asias Avenue, Goudi, 11527 Athens, Greece

## Abstract

Thyroid hormone (TH) is critical for adapting living organisms to environmental stress. Plasma circulating tri-iodothyronine (T3) levels drop in most disease states and are associated with increased oxidative stress. In this context, T3 levels in plasma appear to be an independent determinant for the recovery of cardiac function after myocardial infarction in patients. Thyroid hormone receptor **α**1 (TR**α**1) seems to be crucial in this response; TR**α**1 accumulates to cell nucleus upon activation of stress induced growth kinase signaling. Furthermore, overexpression of nuclear TR**α**1 in cardiomyocytes can result in pathological or physiological growth (dual action) in absence or presence of its ligand, respectively. Accordingly, inactivation of TR**α**1 receptor prevents reactive hypertrophy after myocardial infarction and results in heart failure with increased phospholamban (PLB) expression and marked activation of p38MAPK. In line with this evidence, TH is shown to limit ischemia/reperfusion injury and convert pathologic to physiologic growth after myocardial infarction via TR**α**1 receptor. TR**α**1 receptor may prove to be a novel pharmacological target for cardiac repair/regeneration therapies.

## 1. Introduction

Adaptation to the environmental oxygen variations was an evolutionary challenge and allowed life to evolve in earth. Transition from low to high oxygen environments can increase oxidative stress and result in tissue damage. However, living organisms evolved from aquatic to terrestrial environments by developing mechanisms that enabled adaptation to changes in environmental oxygen. These mechanisms have been evolutionary conserved in mammals allowing mammalian birth to oxygen rich environment or implicated in freeze tolerance and arousal from hibernation [1]. Understanding the molecular basis of the adaptive responses of living organisms to stress may be of physiological relevance in the therapy of diseases. In this context, recent experimental and clinical evidence shows that thyroid hormone (TH) may be critical in stress response and low TH in diseased states is associated with increased oxidative stress [2, 3]. With this evidence in mind, this review highlights the role of thyroid hormone signaling and particularly of thyroid hormone receptor alpha1 (TR*α*1) in cardiac recovery following myocardial injury.

## 2. Adaptation to Environmental Stress: The Role of Thyroid Hormone (TH)

**Figure 1:**
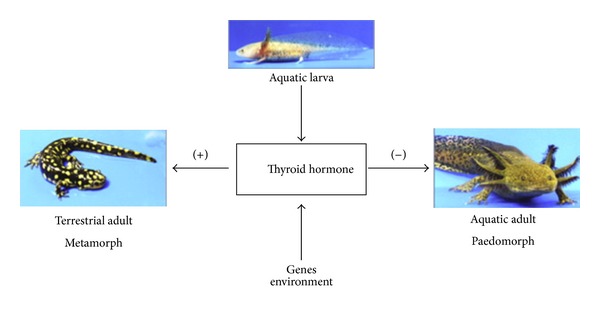
Critical levels of thyroid hormone (TH) induce metamorphosis in salamanders. Low TH can adapt salamander to low oxygen aquatic environment by inducing growth with embryonic characteristics. Addition of TH allows adaptation to terrestrial life and completes metamorphosis. Analogies seem to exist in mammals with TH to determine the phenotypic characteristics of the myocardium (pathological versus physiological growth) after ischemic events. Evolutionary conserved mechanisms of adaptation may be the basis for cardiac repair. (Permission by Johnson and Voss [4].)

Amphibian metamorphosis is the most striking paradigm of adaptation to oxygen rich environment. This biological process is entirely dependent on TH. TH is low during embryonic and early larva development and increases as larva approaches metamorphosis. A similar developmental TH secretion pattern is observed in most species and in humans [4]. Furthermore, distinct changes in deiodinases and thyroid hormone receptors (TRs) expression occur and thus, a single hormone can coordinate responses among different cell types and regulate the temporal sequence of remodeling events during amphibian metamorphosis. More importantly, TH can critically determine the amphibian phenotype (low oxygen, aquatic versus high oxygen, and terrestrial habitats). Thus, in salamanders, low TH results in permanent aquatic habitats, delayed metamorphic timing, and large body size, whereas high TH has opposite effects [4]; see Figure 1.

Environmental stress appears to cause changes in the pattern of TH secretion similar to that observed in the early embryonic stages. This response is likely to be part of an adaptive response of the living organism to environmental stress. Thus, exposure of air breathing perch to water-born kerosene resulted in low TH and unfavorable metabolic changes, while the administration of TH reversed this response [5]. Along this line, cold stunning in sea turtles resulted in undetectable thyroid hormone levels and recovery induced by rewarming was associated with restoration of TH levels [6]. Similarly, in humans, TH levels decline after various stresses including ischemia, infection, and organ failure, but the physiological relevance of this response in regard to post-stress adaptation remains largely unknown [7, 8].

## 3. TH Is Critical for the Recovery after Myocardial Injury

A decline in T3 levels occurs within 48 hours(h) after myocardial infarction (AMI) or 6–24 h after cardiac surgery [9, 10]. Low T3 syndrome is present in nearly 20% of patients with AMI, despite primary percutaneous coronary intervention (PCI). Low fT3 levels are associated with lower survival rate particularly in patients with age less than 75 years [11], indicating that TH may have a role in adapting the heart to myocardial injury. In fact, a link of TH to cardiac recovery after myocardial infarction has been recently established in humans and in experimental studies.

In a series of patients with AMI and primary PCI, left ventricular ejection fraction (LVEF%) 48 hours after the index event was strongly correlated with T3 and not T4 levels in plasma. Furthermore, at 6 months, recovery of cardiac function was correlated with T3 plasma levels and T3 was shown to be an independent determinant of LVEF% recovery [12].

In accordance with this clinical evidence, acute T3 (and not T4) administration after ischemia/reperfusion in isolated rat hearts resulted in significant improvement of postischemic recovery of function [13, 14]. Furthermore, in an experimental model of coronary ligation in mice, cardiac function was significantly decreased and this was associated with a marked decline in T3 levels in plasma. T3 replacement therapy significantly improved the recovery of cardiac function [15, 16].

On the basis of these data, it appears that the active T3 and not T4 is critical for the response to stress. In fact, T4 therapy in patients with euthyroid syndrome due to severe illness was not shown to be beneficial [17, 18].

## 4. TR***α***1 Receptor and Its Physiologic Actions

T3, the active form of TH, exerts many of its actions through its receptors (TRs): TR*α*1, TR*α*2, TR*β*1, and TR*β*2. TRs, with the exception of TR*β*2, are expressed in all tissues and the pattern of expression varies in different types of tissues [19]. TR*α*1 is predominantly expressed in the myocardium and regulates important genes related to cell differentiation and growth, contractile function, pacemaker activity, and conduction [20–22].

The importance of TH in organ maturation during development and its implication in cell differentiation has long been recognized. This unique action seems to be of physiological relevance in stem cell biology and cancer [23, 24]. T3 can promote differentiation of human pluripotent stem cell derived cardiomyocytes (hips-CM) [23] and glioma tumor cell lines [24]. The implication of TR*α*1 in cell differentiation is shown in embryonic myoblast cultures (H9c2), which is considered a suitable model to study cell differentiation. Maturation of H9c2 is TH dependent process [25, 26]. TR*α*1 expression is increased in parallel with the intracellular T3 at the stage of cell differentiation and pharmacological inactivation of TR*α*1 significantly delays cardiac myoblast maturation [27]. Along this line, TR*α*1 is shown to play a critical role in pancreatic *β*-cell replication and in the expansion of the *β*-cell mass during postnatal development [28].

T3 can induce physiologic growth and this action involves the activation of PI3 K/Akt/mTOR pathway. T3 regulates this pathway by the interaction of the cytosol-localized TR*α*1 with the p85*α* subunit of PI3 K [29, 30].

TR*α*1 appears to be required to repress basal expression of *β*-isoform of myosin heavy chain (*β*-MHC) and T3 induced *β*-MHC repression [31]. Deletion of TR*α*1 results in lower levels of *α*-MHC and SERCA mRNA [32], whereas phospholamban (PLB) expression is greater in the myocardium of animals with mutated TR*α*1 [33]. TR*α*1 directly binds at the PLB promoter region. T3 can trigger alterations in covalent histone modifications at the PLB promoter which are associated with gene silencing with lower histone H3 acetylation and histone H3 lysine 4 methylation [34]. In line with this evidence, contractile dysfunction is a consistent observation in all studies using animals with mutated or deleted TR*α*1 receptor [32, 33].

TH regulates the transcription of pacemaker channel genes such as HCN2 and HCN4 and this action involves TR*α*1 receptor [32]. Deletion of TR*α*1 results in bradycardia [32, 35]. TR*α*1 is also shown to bind to an element of rat connexin 43 promoter region which may be of physiological relevance regarding electrical conduction [36].

TH can control glucose metabolism in the heart via TR*α*1 receptor. Thus, glucose utilization in the myocardium is impaired in mice with mutated TR*α*1 [37]. Furthermore, pharmacological inhibition of TR*α*1 in rats resulted in increased glycogen content in the myocardium [38].

TR*α*1 is the predominant TR isoform in mouse coronary smooth muscle cells (SMCs) and seems to have a regulatory role in the coronary artery contractile function. Coronary SMCs from TR*α*1 knock-out mice exhibit a significant decrease in K^+^ channel activity. Furthermore, in those arteries, vascular contraction is significantly enhanced [39].

Collectively, it appears that TR*α*1 has a regulatory role in cardiac homeostasis and thus, it is likely to be implicated in the pathophysiology of cardiac disease. This hypothesis has not, until recently, been explored.

## 5. TR***α***1 and Response of the Myocardium to Stress

The potential link of TH signaling to cardiac pathology and particularly of TR*α*1 receptor has been investigated in several studies with much controversy surrounding this issue. Initial observations showed that TR*α*1 mRNA is suppressed in left ventricles of patients with dilated cardiomyopathy in comparison with donor hearts [40]. Accordingly, TR*α*1 mRNA was found to be downregulated in the myocardium of animals with ascending aortic constriction (TAC) [41, 42]. Furthermore, TR*α*1 mRNA was found to be suppressed after phenylephrine (PE, an alpha1-adrenergic agonist, which is a stimulus for pathologic growth) administration in neonatal cardiomyocytes [42]. Overexpression of TR*α*1 was shown to reverse PE and TAC induced hypertrophic phenotype [41, 42]. However, this was not a consistent result in all studies. Overexpression of TR*α*1 resulted in physiologic growth in one study [42] and pathologic growth in another study [43]. Here it should be noted that, in all those studies, TR*α*1 was measured at mRNA level and not at protein level. TR*α*1 protein expression was measured in subsequent studies in cardiac specimens from patients with heart failure. TR*α*1 was found to be upregulated in one study [44] and downregulated in another study [45]. To add to the controversy, TR*α*1 was shown to be overexpressed [46] or downregulated [47] in animal models of cardiac remodeling after myocardial infarction. On the basis of this conflicting evidence, it is conceivable that clear conclusions cannot be drawn regarding potential role of TR*α*1 receptor in stressed myocardium.

## 6. TR***α***1: A Component of Stress Induced Growth Signaling Pathways

**Figure 2:**
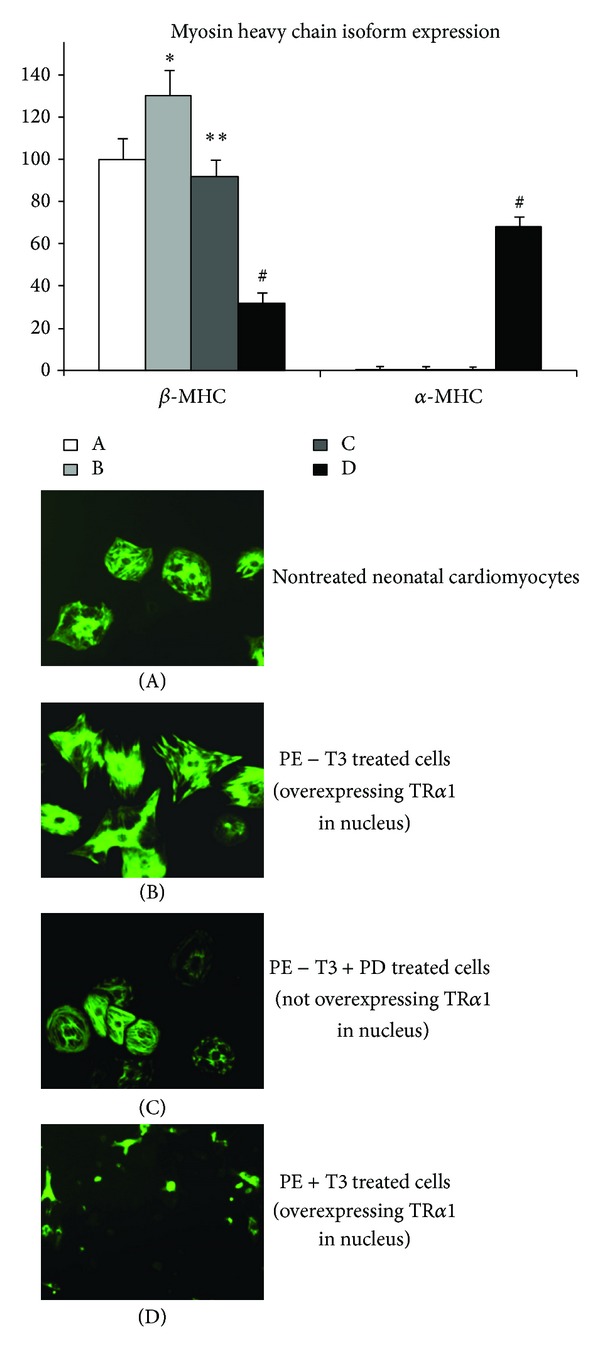
Thyroid hormone (TH) determines the growth response to stress. Stress induced (by PE, a growth stimulus) overexpression of TR*α*1 in neonatal cardiomyocytes resulted in pathologic growth with dominant beta-MHC expression only in the absence of TH in the cultured medium (PE-T3) (B). This response was abolished after PD98059 administration (an ERK inhibitor) which prevents PE induced TR*α*1 accumulation in nucleus (PE-T3 + PD) (C). In the presence of TH in cultured medium, PE induced TR*α*1 accumulation in nucleus resulted in physiologic growth with suppressed beta-MHC and increased alpha-MHC (PE + T3) (D) *P < 0.05 versus A, **P < 0.05 versus B, ^#^ *P* < 0.05 versus A, B, and C, PE = phenylephrine, MHC = myosin heavy chain.

Recent experimental studies have shed more light regarding the role of TR*α*1 in the response of the myocardium to stress and seem to resolve the controversy. Thus, a distinct pattern of TR*α*1 expression is shown to occur in the myocardium after acute myocardial infarction, indicating a potential link of TR*α*1 to reactive cardiac hypertrophy. TR*α*1 (nuclear part) was shown to be upregulated during the development of compensatory pathological hypertrophy in parallel with a greater activation of ERK and mTOR growth signaling. Consequently, TR*α*1 declines along with a marked reduction in ERK and mTOR signaling activation on the transition of pathological hypertrophy to congestive heart failure [48]. Studies in cultured cardiomyocytes further showed that TR*α*1 receptor can be overexpressed in cell nucleus in response to growth stimuli such as phenylephrine (PE) [27]. This response was shown to be due to redistribution of TR*α*1 from cytosol to nucleus. This process is regulated via ERK and mTOR signaling. In those experiments, overexpression of TR*α*1 receptor was shown to be associated with pathological growth (with dominant *β*-MHC expression) only in the absence of TH in culture medium Figure 2. Furthermore, inhibition of ERK and mTOR signaling abolished TR*α*1 accumulation in cell nucleus and prevented the development of PE induced pathological growth; see Figure 2. This response could be elicited by *α*1 adrenergic and not *β*2-adrenergic stimulation (unpublished data) while treatment of neonatal cells with inflammatory mediators, such as TNF-alpha, had no effect on nuclear TR*α*1 expression [49]. Collectively, these data provide substantial evidence that stress induced accumulation of TR*α*1 in cell nucleus may be an important component of the mechanisms involved in compensatory growth response after myocardial infarction. This hypothesis has recently been tested in studies in which debutyl-dronedarone (DBD), a TR*α*1 inhibitor, was administered after AMI in mice [50]. DBD treatment was shown to reduce recovery of cardiac function, prevent compensatory hypertrophy, increase PLB expression (TR*α*1 responsive gene), and result in marked activation of p38 MAPK [51]. The latter may be of important physiological relevance. Stress induced activation of p38 MAPK can cause apoptosis, low proliferative activity, and impaired tissue repair/regeneration [52–54].

## 7. TR***α***1: A Molecular Switch to Convert Pathologic to Physiologic Growth

The potential link of TR*α*1 to growth response has been revealed in neonatal cardiomyocytes cultures in which phenylephrine (PE) was administered in the presence or absence of TH in culture medium. In this series of experiments, PE administration resulted in increased nuclear TR*α*1 content and in pathologic growth (dominant *β*-MHC expression) in the absence of T3 and physiologic growth in the presence of T3 in culture medium [27]; see Figure 2. Thus, TR*α*1 receptor appears to act as a molecular switch to convert pathologic to physiologic growth. Consistent with this evidence, TH replacement therapy following myocardial infarction in mice resulted in compensatory hypertrophy with adult pattern of myosin isoform expression [16]. Furthermore, increased expression of liganded TR*α*1 in the myocardium after physical training in patients with heart failure and mechanical support devices was associated with upregulation of physiologic growth kinase signaling [55]. Similarly, TH restored myelination and clinical recovery after intraventricular hemorrhage by converting the unliganded, aporeceptor TR*α*1 to holoreceptor [56].

## 8. TR***α***1 and Ischemia/Reperfusion Injury

TH has long been considered to be detrimental for the response of the myocardium to ischemic stress. However, this long standing belief has been challenged over the past years. In fact, in a series of studies using isolated rat heart models of ischemia/reperfusion, TH pretreatment was shown to be beneficial and mimic the effect of ischemic preconditioning [57]. Furthermore, T3 (and not T4) administration at reperfusion suppressed apoptosis, limited necrosis, and improved postischemic recovery of function [13, 14]. Similarly, TH treatment after myocardial infarction limited infarct size [58] and reduced apoptosis in the border zone of the infarcted area [59]. The reparative effect of TH seems to be mediated via activation of prosurvival signaling pathways. Thus, TH activates Akt [16, 59–61] and regulates PKC isoforms expression [62, 63], HSP70 expression [64], and HSP27 expression and phosphorylation and translocation [65]. Furthermore, TH suppresses ischemia/reperfusion induced p38 MAPK and JNK activation [14, 66]. TH reparative action is shown to be mediated via TR*α*1 receptor [13]. Here, it is worth mentioning that T3 can also limit streptozotocin (STZ) induced beta pancreatic cell apoptosis via TR*α*1 receptor. Thus, TH administration in STZ treated animals with myocardial infarction resulted in increased insulin levels in plasma and significant improvement of the postischemic cardiac dysfunction [61].

## 9. Clinical and Therapeutic Implications

Reperfusion injury and postischemic cardiac remodeling remain still a therapeutic challenge in the management of patients with heart disease [67, 68]. The discovery of novel pharmacological targets such as TR*α*1 receptor may be of important clinical and therapeutic relevance. TH has already been tried in clinical settings of controlled ischemia/reperfusion, such as CABG or heart donors preservation. Thus, T3 treatment postoperatively limited reperfusion injury and improved haemodynamics in patients undergoing CABG [69]. Furthermore, T3 treatment initiated one week before GABG resulted in improved cardiac index and reduced requirements of inotropes [70]. Similarly, TH has been used in heart donors to increase the probability of success in donor organ transplantation. However, a clear benefit of this treatment has not been demonstrated [71]. This may be due to the fact that most of the patients were receiving T4 instead of the active T3 and TH treatment was used in nonischemic stable donor hearts. In fact, when T3 was administered in a series of 22 unstable (ischemic) heart donors (considered unsuitable for transplantation), 17 of those patients progressed to successful transplantation [72]. Here it should be noted that TH was shown to facilitate recovery in patients with end-stage heart failure and mechanical support devices [73].

On the basis of these preliminary clinical data, large-scale clinical trials may be needed to demonstrate the beneficial effect of TH in clinical settings of ischemia/reperfusion. Furthermore, the recognition that TH can mediate important physiological and pharmacological actions via TR*α*1 receptor may allow selective pharmacological manipulation of TH signaling via TR*α*1 agonists. Currently, only TR*β* analogs have been synthesized to control cholesterol metabolism. However, a chemical compound (CO23) which is assumed to be a TR*α*1 selective agonist has recently been synthesized. This compound, although it was shown to be selective for TR*α*1 receptor in amphibian models, it lost its selectivity in rat [74, 75]. This may be due to differences in TR expression in developing and mature tissues. This issue is of important therapeutic relevance and merits further investigation.

## 10. Concluding Remarks

**Figure 3:**
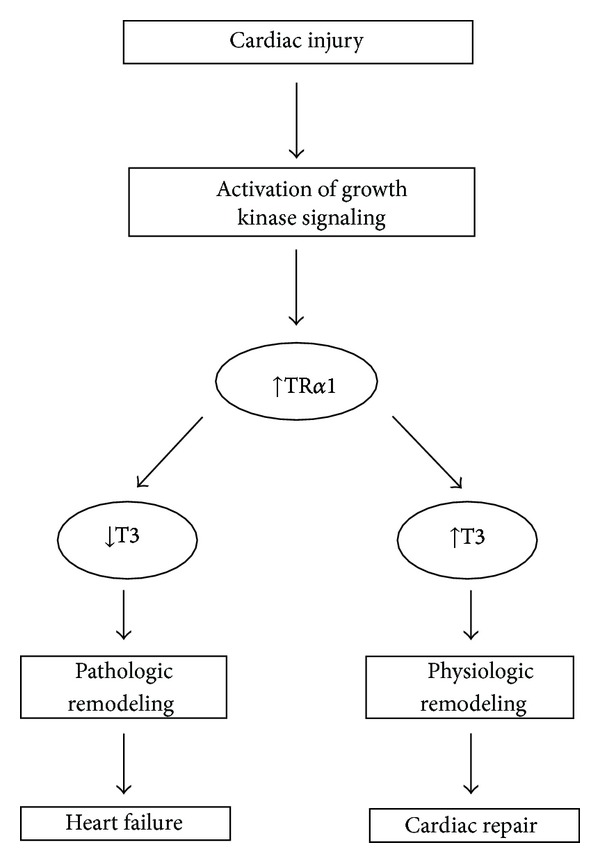
Schematic showing TR*α*1 involvement in the response of the myocardial tissue to injury.

TH is long known to be critical in organ maturation and regulation of metabolism. However, recent accumulating evidence shows that TH is crucial for the response of living organisms to environmental stress. In particular, TR*α*1 receptor seems to be an important determinant for the reactive growth response which occurs after myocardial injury. TR*α*1 can act as a molecular switch to convert pathological to physiologic growth; see Figure 3. Due to this dual action, TH, via TR*α*1 receptor, can limit myocardial injury and rebuild the injured myocardium. It is likely that TR*α*1 receptor may prove a novel pharmacological target for cardiac repair/regeneration.
